# Supplementary material for: A computational model of shared fine-scale structure in the human connectome
Source: PLoS Comput Biol. 2018 Apr 17;14(4):e1006120. doi: 10.1371/journal.pcbi.1006120 (PMC5922579; doi:10.1371/journal.pcbi.1006120)
Supplement: S1 Text — (DOCX) [file pcbi.1006120.s001.docx]

**S1 Text.  Overview of supplemental figures and tables**

We compared the common model of information spaces based on CHA to a common model based on response hyperalignment (RHA)[16].  Comparison of the effects of CHA and RHA on ISC of representational geometry and bsMVPC of movie time-segments showed that, whereas alignment of connectivity profiles was slightly better after CHA than after RHA (S1 Fig),  alignment of patterns of response was slightly better after RHA than after CHA (S2 Fig and S3 Fig). We also illustrate the fine-grain structure in group mean connectivity patterns after CHA (Figure 7), and in S4 Fig we show all individual connectivity patterns in the same left lateral-occipital/inferior-temporal cortical field with connectivity targets in left mid fusiform and mid superior temporal sulcus cortices.  S1 Table shows the coordinates used as the centers of functional ROIs used to quantify results in Figures 2, 3, and 4, S2 Table lists the tasks and task contrasts from the HCP database used in Figure 6.
