# Supplementary figures and images for: A computational model of shared fine-scale structure in the human connectome
Source: PLoS Comput Biol. 2018 Apr 17;14(4):e1006120. doi: 10.1371/journal.pcbi.1006120 (PMC5922579; doi:10.1371/journal.pcbi.1006120)

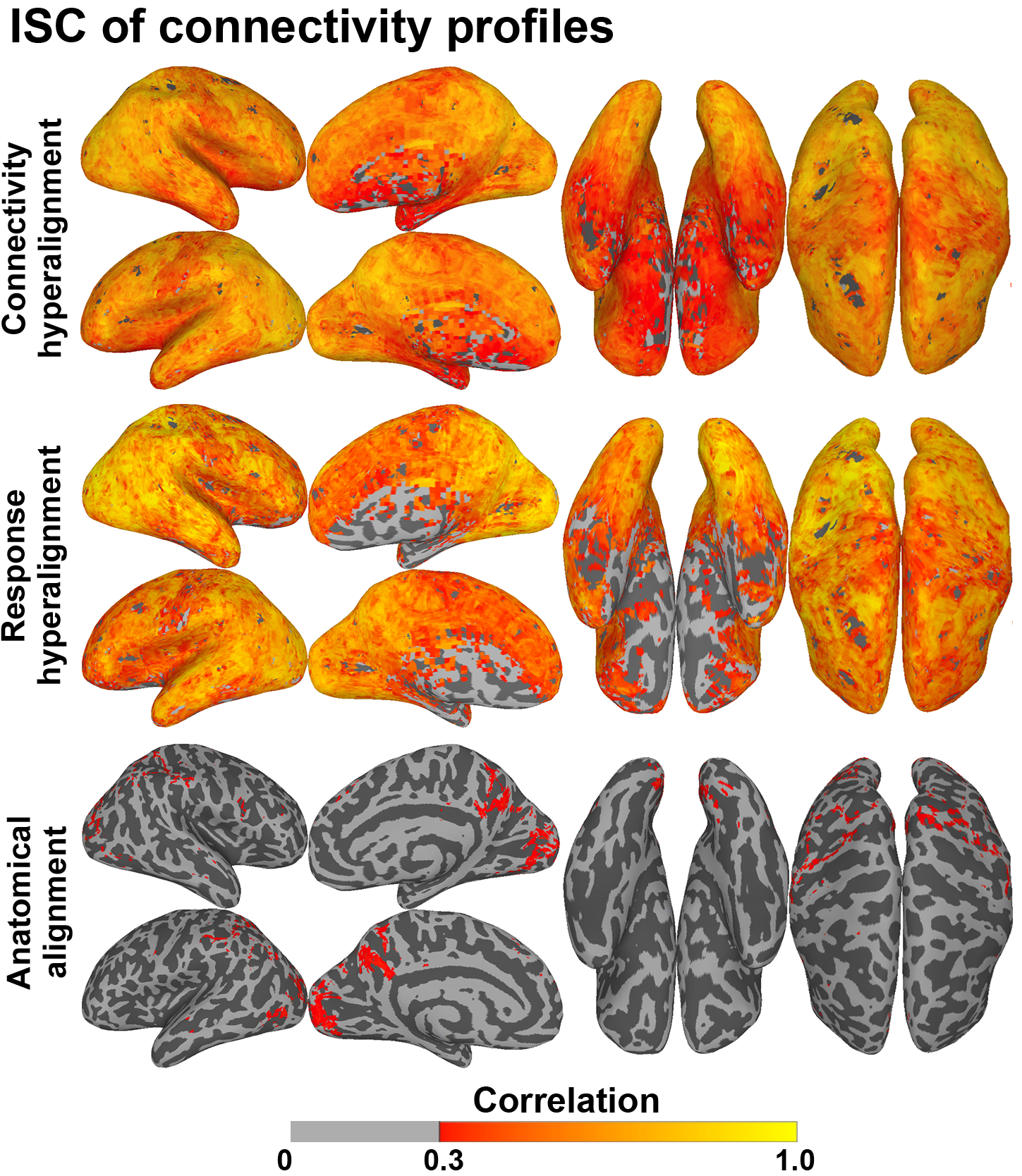

Supplement: S1 Fig — ISCs of representational geometry in each voxel are mapped onto cortical surfaces after CHA, RHA, and anatomical alignment, RHA, and CHA. Maps of ISCs after CHA and anatomical alignment are identical to maps shown in Fig 2 and are reproduced here to facilitate comparison to RHA. (TIF) [file pcbi.1006120.s002.tif]

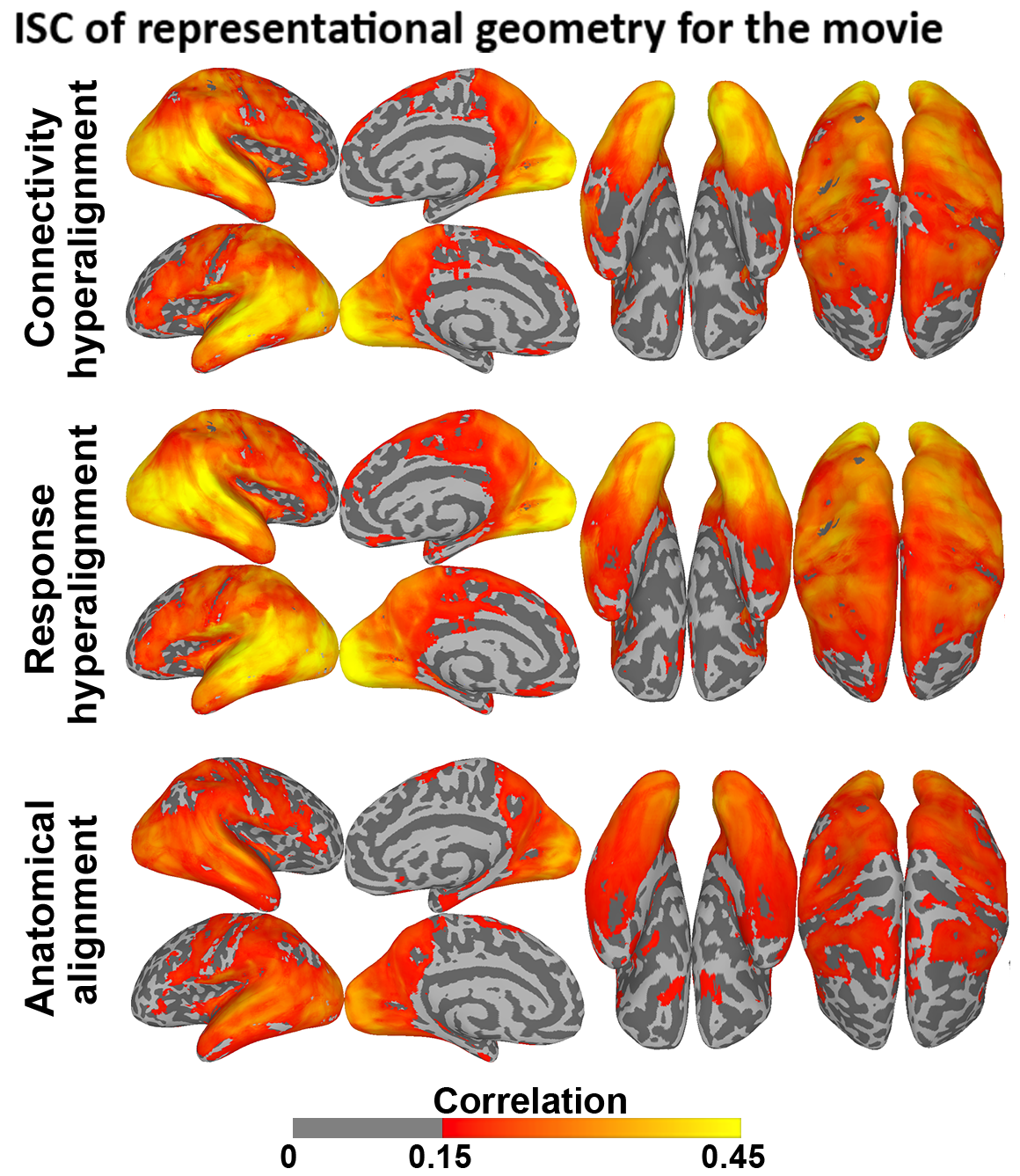

Supplement: S2 Fig — ISCs of representational geometry in each voxel are mapped onto cortical surfaces after CHA, RHA, and anatomical alignment, RHA, and CHA. Maps of ISCs after CHA and anatomical alignment are identical to maps shown in Fig 5 and are reproduced here to facilitate comparison to RHA. (TIF) [file pcbi.1006120.s003.tif]

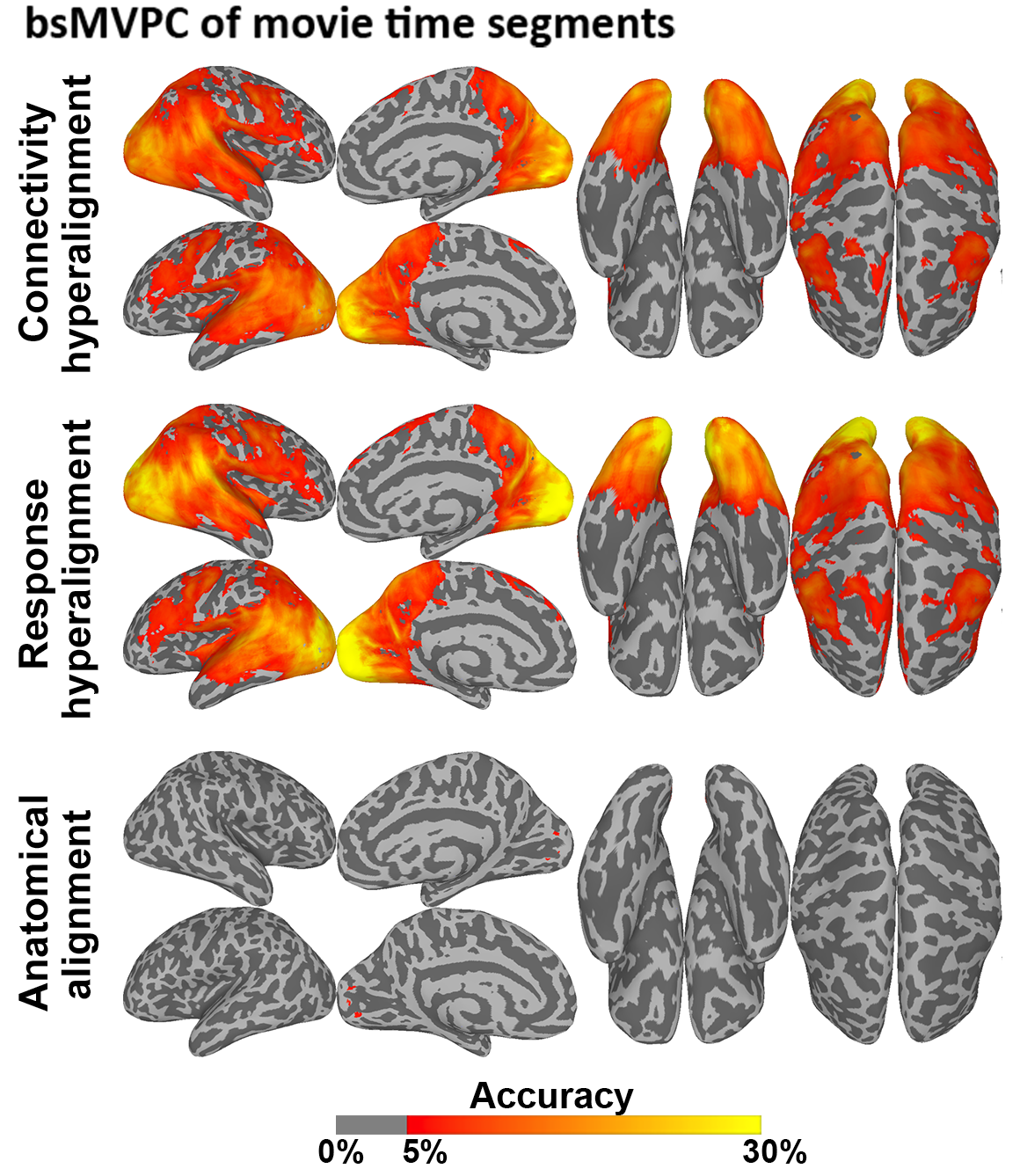

Supplement: S3 Fig — Classification accuracies in each searchlight mapped on cortical surfaces after CHA, RHA, and anatomical alignment. (TIF) [file pcbi.1006120.s004.tif]

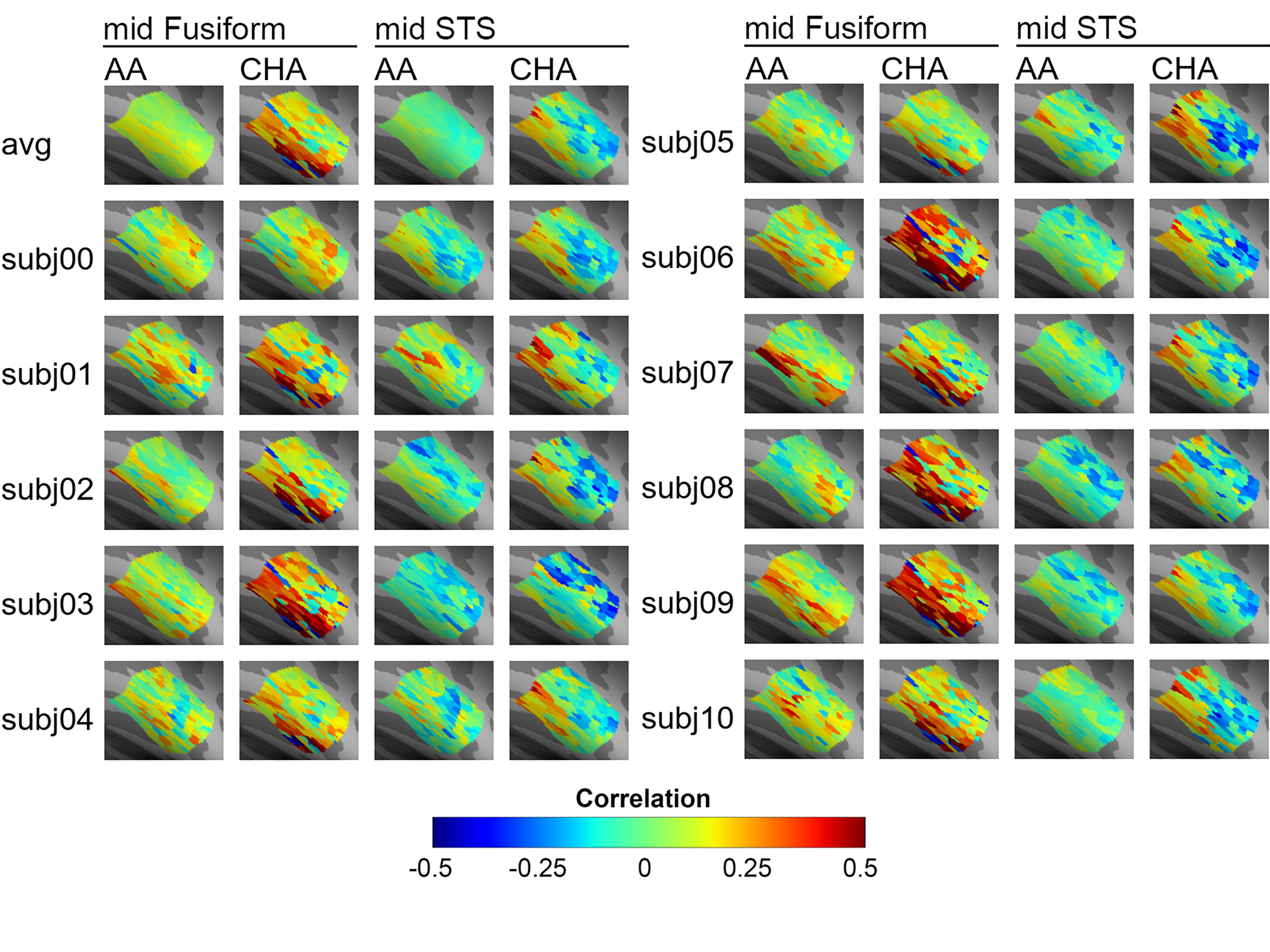

Supplement: S4 Fig — Connectivity patterns are shown for anatomically-aligned data and data transformed into the common model connectome. Connectivities are correlations between time series responses to the movie. CHA transformation matrices were derived from independent data from the other movie half. Group mean connectivity patterns are shown in the first row for comparison (also shown in Fig 8). (TIF) [file pcbi.1006120.s005.tif]
