# Supplementary material for: A computational model of shared fine-scale structure in the human connectome
Source: PLoS Comput Biol. 2018 Apr 17;14(4):e1006120. doi: 10.1371/journal.pcbi.1006120 (PMC5922579; doi:10.1371/journal.pcbi.1006120)
Supplement: S1 Table — (DOCX) [file pcbi.1006120.s006.docx]

**S1 Table.** Selected cortical loci implicated in visual, auditory, cognitive, and social functions from Neurosynth.

| **Search Term** | **Hemisphere** | **MNI Coordinates (x, y, z)** | | |
| --- | --- | --- | --- | --- |
| **Visual** | | | | |
| V1 | Left | -4 | -82 | -4 |
|  | Right | 10 | -94 | 2 |
| MT | Left | -42 | -72 | 2 |
|  | Right | 44 | -66 | 2 |
| Visual word form area | Left | -46 | -60 | -14 |
| FFA | Left | -42 | -52 | -20 |
|  | Right | 40 | -50 | -22 |
| Scenes (PPA) | Left | -26 | -46 | -10 |
|  | Right | 34 | -38 | -12 |
| **Auditory** | | | | |
| Primary Auditory (A1) | Left | -44 | -30 | 10 |
|  | Right | 52 | -14 | 4 |
| Voice | Left | -60 | -14 | 0 |
|  | Right | 60 | -4 | -12 |
| Music | Left | -52 | -14 | 0 |
|  | Right | 60 | -20 | 4 |
| **Cognitive** | | | | |
| Calculations | Left | -30 | -66 | 38 |
|  | Right | 34 | -64 | 44 |
| Broca’s area | Left | -52 | 14 | 12 |
| Working memory | Left | -44 | 26 | 24 |
|  | Right | 44 | 42 | 26 |
| **Social** | | | | |
| MPFC |  | 6 | 54 | 14 |
| TPJ | Left | 50 | -52 | 22 |
|  | Right | -52 | -56 | 22 |
| Precuneus |  | 0 | -56 | 40 |

V1 - primary visual cortex, MT - middle temporal visual motion area, FFA - fusiform face area, PPA - parahippocampal place area, MPFC - medial prefrontal cortex, TPJ - temporoparietal junction.

**S2 Table.** Task maps used from the HCP data.

| **Experiment/Task** | **Maps** |
| --- | --- |
| MOTOR | CUE, LF, LH, RF, RH, T, CUE-AVG, LF-AVG, LH-AVG, RF-AVG, RH-AVG, T-AVG |
| LANGUAGE | MATH, STORY, MATH-STORY |
| WM (Working Memory) | 2BK_BODY, 2BK_FACE, 2BK_PLACE, 2BK_TOOL, 0BK_BODY, 0BK_FACE, 0BK_PLACE, 0BK_TOOL, 2BK, 0BK, 2BK-0BK, BODY, FACE, PLACE, TOOL, BODY-AVG, FACE-AVG, PLACE-AVG, TOOL-AVG |
| RELATIONAL | MATH, REL, MATCH-REL |
| EMOTION | FACES, SHAPES, FACES-SHAPES |
| SOCIAL | RANDOM, TOM, TOM-RANDOM |
| GAMBLING | PUNISH, REWARD, PUNISH-REWARD |

LF - Left Finger, RH - Right Hand, 2BK - Two-Back, TOM - Theory of Mind
