# Supplementary material for: A computational model of shared fine-scale structure in the human connectome
Source: PLoS Comput Biol. 2018 Apr 17;14(4):e1006120. doi: 10.1371/journal.pcbi.1006120 (PMC5922579; doi:10.1371/journal.pcbi.1006120)
Supplement: S2 Table — (DOCX) [file pcbi.1006120.s007.docx]

**S2 Table.** Task maps used from the HCP data.

| **Experiment/Task** | **Maps** |
| --- | --- |
| MOTOR | CUE, LF, LH, RF, RH, T, CUE-AVG, LF-AVG, LH-AVG, RF-AVG, RH-AVG, T-AVG |
| LANGUAGE | MATH, STORY, MATH-STORY |
| WM (Working Memory) | 2BK_BODY, 2BK_FACE, 2BK_PLACE, 2BK_TOOL, 0BK_BODY, 0BK_FACE, 0BK_PLACE, 0BK_TOOL, 2BK, 0BK, 2BK-0BK, BODY, FACE, PLACE, TOOL, BODY-AVG, FACE-AVG, PLACE-AVG, TOOL-AVG |
| RELATIONAL | MATH, REL, MATCH-REL |
| EMOTION | FACES, SHAPES, FACES-SHAPES |
| SOCIAL | RANDOM, TOM, TOM-RANDOM |
| GAMBLING | PUNISH, REWARD, PUNISH-REWARD |

LF - Left Finger, RH - Right Hand, 2BK - Two-Back, TOM - Theory of Mind
